# Impact of high-speed nanodroplets on various pathogenic bacterial cell walls

**DOI:** 10.1128/jb.00139-24

**Published:** 2024-10-09

**Authors:** Yurina Tamura, Masato Kawamura, Takehiko Sato, Tomoki Nakajima, Siwei Liu, Takumi Sato, Shigeru Fujimura

**Affiliations:** 1 Division of Clinical Infectious Diseases & Chemotherapy, Tohoku Medical and Pharmaceutical University, Graduate School of Pharmaceutical Sciences, Sendai, Japan; 2 Institute of Fluid Science, Tohoku University, Sendai, Japan; University of Southern California, Los Angeles, California, USA

**Keywords:** nanodroplet, disinfection, biofilm, collision

## Abstract

Although the development of disinfection technologies with novel mechanisms has stagnated, we demonstrate the bactericidal effects and mechanisms of high-speed nanodroplet generation technology. The first development of this technology in 2017 gushes out a water droplet of 10 nm in size at 50 m/s; however, the target surface does not become completely wet. Nanodroplets were exposed to biofilm models of *Staphylococcus aureus*, *Pseudomonas aeruginosa*, *Escherichia coli*, and *Serratia marcescens*. This phenomenon was verified when the nanodroplets collide with the surface of the bacteria at an impact pressure of ~75 MPa. *S. aureus* was exposed to nanodroplets for 30 seconds at 75 MPa, which exploded the bacterial body and completely sterilized. Eighteen MPa damaged the bacterial surface, causing peptidoglycan leakage. *S. aureus* was repaired and survives in this state. In contrast, in Gram-negative bacteria, nanodroplets with 18 MPa penetrated some biofilm-forming bacteria but did not hit all of them, and the viable count was not significantly reduced. Although all three bacterial species were completely sterilized at 75 MPa, the disinfectant effect was affected by the biomass of the biofilm formed. In summary, our findings prove that nanodroplets at 18 MPa on the bacterial surface were ineffective in killing bacteria, whereas at 75 MPa, all four bacterial species were completely sterilized. The disinfection mechanism involved a high-velocity collision of nanodroplets with the bacteria, physically destroying them. Our results showed that disinfection using this technology could be an innovative method that is completely different from existing disinfection techniques.

**IMPORTANCE:**

Although existing disinfection techniques demonstrate bactericidal effects through chemical reactions, concerns regarding human toxicity and environmental contamination have been raised. To the best of our knowledge, this study is the first in the world to reveal that the use of this technology, with nanodroplets of less than 100 nm, can destroy and sterilize bacterial cells by colliding with biofilm-forming bacteria at 75 MPa. Furthermore, because this technology uses only water, it can solve the problems of human toxicity and environmental contamination caused by existing disinfection techniques. Because of its minimal water usage, it can be employed for sanitation worldwide without being limited to specific regions. Our report proposes an unprecedented physical disinfection approach that utilizes a high-speed nanodroplet generation technology.

## INTRODUCTION

Since Koch reported that infectious diseases were caused by microorganisms in 1876, the development of disinfectants and research on their bactericidal effects have progressed since the 1890s, and heat treatment was invented (1, 2). In 1901, the bactericidal effects of ultraviolet (UV) lamps were reported (3). In recent years, numerous attempts have been made to improve or combine existing disinfection techniques to kill pathogenic microorganisms (4–8). The accumulation of these studies will facilitate the selection and utilization of disinfection techniques according to our needs. However, despite advances in science and technology, disinfection techniques with novel mechanisms have not been developed for more than 120 years since the bactericidal effects of UV were revealed.

Existing disinfection methods may not be effective because most bacteria can protect their bodies from external stimuli by forming a biofilm (9–13). It has also been reported that the continuous exposure of bacteria to disinfectants decreases their susceptibility to disinfectants and antimicrobial agents (14, 15). Furthermore, bacteria exhibit adaptive resistance to UVC irradiation (16, 17). It is assumed that bacteria will continue to evolve and become increasingly resistant to the existing disinfection techniques. Therefore, the development of novel antiseptic techniques is essential.

**Fig 1:**
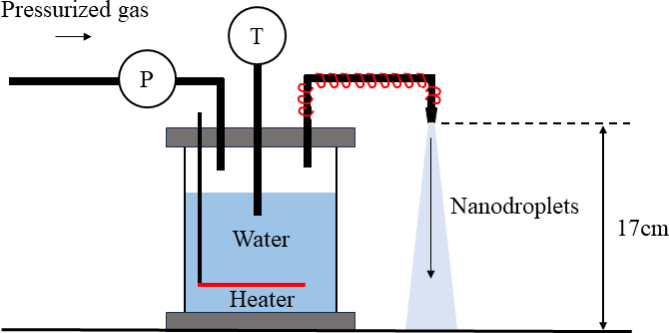
High-speed nanoscale mist-generating device. P: denotes the pressure gauge, T: denotes the temperature meter. Distance from he nozzle to desk is 17 cm. The internal pressure in the steam generator: five atm, water temperature: 152.3°C, nozzle temperature: 175°C, velocity of the nanodroplet exposed from the nozzle: ~50 m/s, nanodroplet temperature: ~30°C.

Recently, Zare RN *et al.* showed that microdroplets (10 µm in diameter) have a chemical sterilizing effect through the generation of reactive oxygen species, including OH radicals and hydrogen peroxide (18). Moreover, it was also shown that hydrogen peroxide is generated during microdroplet formation when electrons are desorbed on the droplet surface and OH is generated and combined (19). We also newly developed high-speed nanodroplet generation technology using only water (20) and examined the bactericidal effect of this technology. This is the world’s first technology in which water droplets of less than 100 nm can gush out at a velocity of ~50 m/s at a distance of 5 cm from the exposure nozzle, using water vapor condensation (Fig. 1); it could continuously gush out for 25 minutes with only 300 mL of water and does not wet the target surface. The size of the droplet diameter can be inferred from the diameter of the droplet traces formed on the surface of the cleaning process indicator. Numerous micropores are formed on the surface of the cleaning process indicator by the treatment of nanodroplets. The diameter of these pores is approximately 300 nm. On the other hand, the experimental study by Uehara *et al.* (21) on the microdroplet impact shows that the diameter of the droplet spreading by the impact on the water film is roughly four times larger. In other words, it can be inferred that the nanodroplet diameter in this study is less than 100 nm. Although extremely small amounts of OH radicals and hydrogen peroxide as well as microdroplets were detected in this study, none of them were at the concentrations that would allow sterilization. Therefore, we focused on the phenomenon that occurs when nanodroplets collide with bacteria. There are several types of existing disinfection techniques; however, these disinfection mechanisms include chemical reactions between disinfectants and bacterial cell membranes, enzyme proteins or nucleic acids, heat-induced protein denaturation, UV-induced nucleic acid mutations, and oxidative damage (7, 22–26). In other words, no technique involves killing bacteria by the collision of water nanodroplets.

This study aimed to elucidate the bactericidal effects and mechanisms of exposure of various bacteria to high-speed nanodroplets.

## RESULTS

### Quantification of bacterial biofilm biomass

The bacterial biofilm biomass on the contact lens was quantified using a crystal violet assay. The absorbance values of the biofilms formed by *Escherichia coli*, *Serratia marcescens*, and *Staphylococcus aureus* were 0.33, 0.22, and 0.31, respectively. In contrast, the absorbance of the *Pseudomonas aeruginosa* biofilm was 1.86, which was six to nine times higher than that of the three aforementioned bacterial species.

### Viable count after exposure to nanodroplets

**Fig 2:**
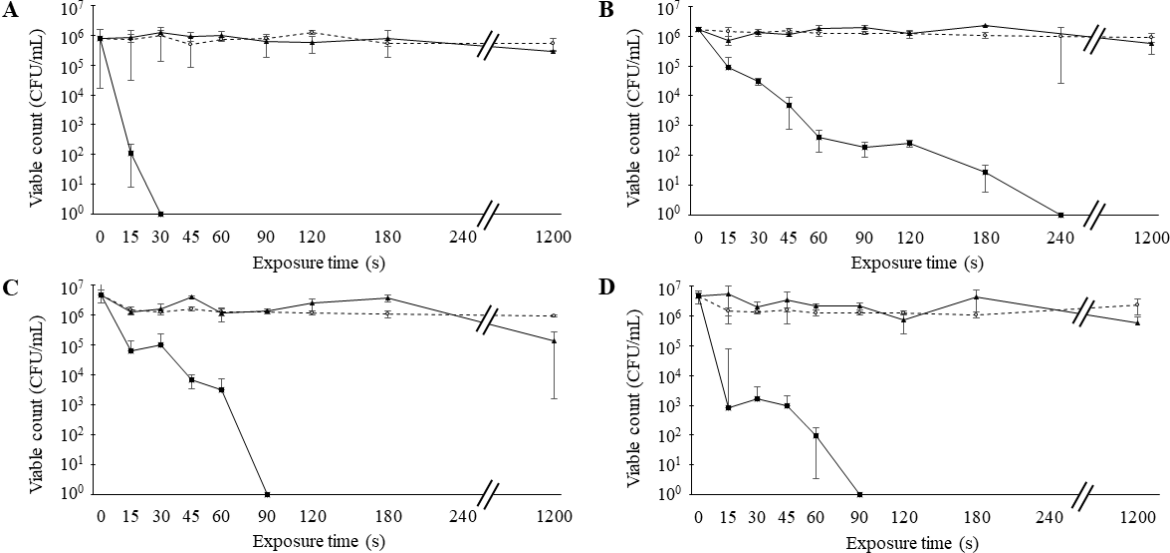
Changes in viable count over time. The viable counts of *S. aureus* (**A**), *P. aeruginosa* (**B**), *E. coli* (**C**), and *S. marcescens* (**D**) were measured after exposure to nanodroplets at a distance of 17 cm or 5 cm for each time. Triangles and squares indicate viable count after exposure to nanodroplets at a distance of 17 cm or 5 cm, respectively, and circles indicate control.

When biofilm-forming bacteria were exposed to nanodroplets at a distance of 17 cm from the nozzle exit, significant decrease in the viable count was not observed for any four bacterial species until 20 minutes later (Fig. 2). At this time, the dispersal of viable bacteria to the biofilm model surroundings was not observed. However, biofilm-forming *S. aureus* was completely sterilized at a distance of 5 cm after exposure for 30 seconds (Fig. 2A). In contrast, the time to complete the sterilization of biofilm-forming Gram-negative bacteria was 90 seconds for *E. coli* and *S. marcescens* and 240 seconds for *P. aeruginosa* (Fig. 2B through D). After each exposure time, a total average of 1.25 × 10^3^ CFU of viable bacteria was observed in each of the four bacterial species up to a maximum distance of 10 cm from the biofilm model.

In planktonic cells, the viable count of *S. aureus* did not decrease after 20 minutes of exposure at a distance of 17 cm, similar to when the biofilm was formed (Fig. S1A). At the same distance, 10^1^–10^2^ CFU/mL of each three Gram-negative bacteria was sterilized after 20 minutes of exposure (Fig. S1B through D), and at a distance of 5 cm, all four bacterial species were completely sterilized in a shorter time than when the biofilm was formed (Fig. S1).

### Mechanism of sterilization by exposure to nanodroplets

**Fig 3:**
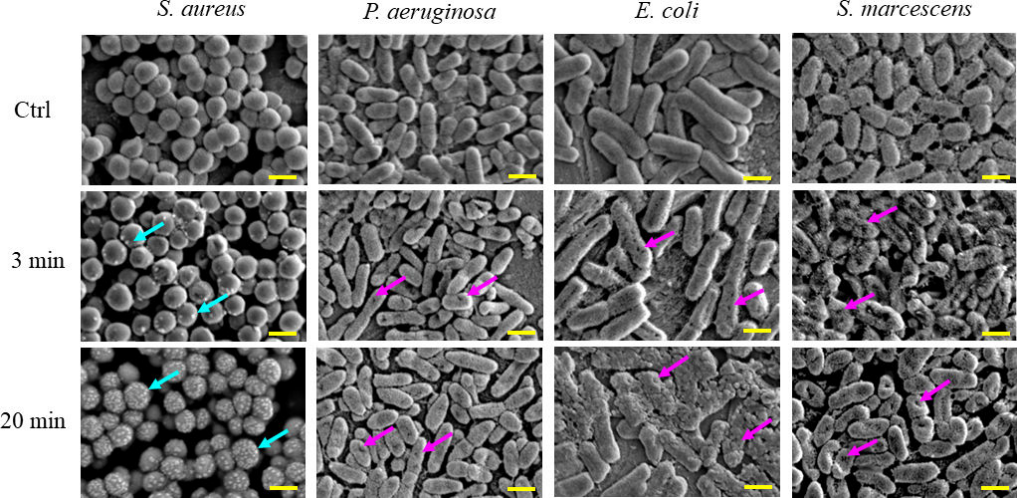
SEM images of the surface of each bacterium after exposure to nanodroplets at a distance of 17 cm. After 3 minutes of exposure, protuberances (light blue arrows) appeared on the surface of *S. aureus*, and the number of projections increased after 20 minutes of exposure. Three Gram-negative bacteria showed multiple holes after 3 minutes of exposure (pink arrows). Bar, 1 µm, magnification: ×15,000.

**Fig 4:**
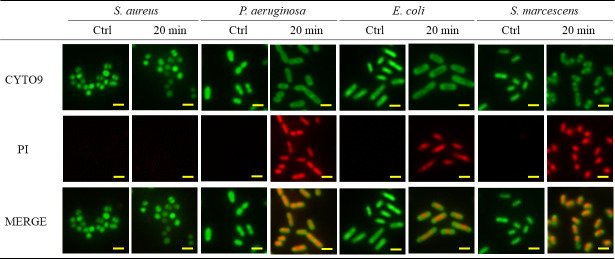
LIVE/DEAD stained images of four biofilm-forming bacteria after exposure to nanodroplets at a distance of 17 cm for 20 minutes. If stained for PI, the cell membrane was considered damaged. Bar, 2 µm, magnification: ×100.

**Fig 5:**
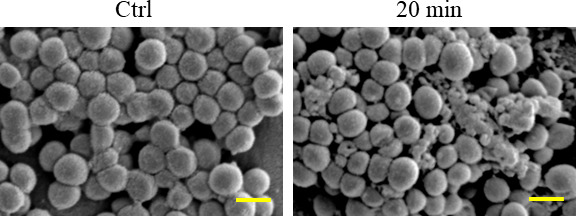
SEM image of *S. aureus* cultured on MHA for 24 hours after exposure to nanodroplets at a distance of 17 cm for 20 minutes. Bar, 1 µm, magnification: ×15,000.

**Fig 6:**
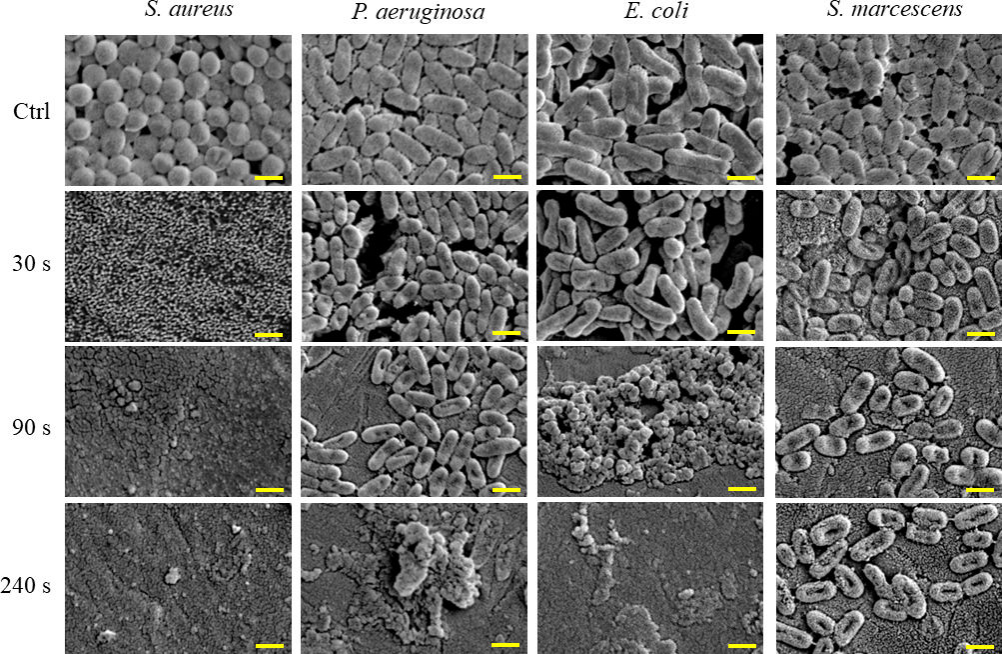
SEM images of four bacterial species of biofilm-forming bacteria after exposure to nanodroplets at a distance of 5 cm. After 30 seconds of exposure to nanodroplets, it was assumed that the high-speed collision of the nanodroplets triggered an explosion of *S. aureus* with a turgor pressure of 20 atm. Therefore, the bacteria did not keep their shape after 30 seconds of exposure, and what was observed by the SEM image was considered to be a fragment of the bacterial cells. In Gram-negative bacteria, the formation of holes on the bacterial surface and the destruction of the bacterial body were observed over time. Bar, 1 µm, magnification: ×15,000.

When biofilm-forming *S. aureus* was exposed to nanodroplets at a distance of 17 cm for 3 minutes, projections with a diameter of approximately 100 nm appeared on the bacterial surface, and numerous projections were observed after 20 minutes of exposure (Fig. 3; Fig. S2). *S. aureus* in this state was not stained by propidium iodide (PI) (Fig. 4). In addition, the luminance of calcein-loaded *S. aureus* was 81 ± 13 cd/m^2^. When the nanodroplets were exposed to this at a distance of 17 cm for 20 minutes, the luminance was 78 ± 17 cd/m^2,^ and leakage of calcein was not observed (Fig. S3). Therefore, at this time, there were numerous projections on the cell wall of *S. aureus*, but no membrane damage was found. After incubation on Mueller–Hinton agar (MHA) at 35°C for 24 hours, some cells exhibited destruction (i.e., irregular shapes), while most reverted to the normal bacterial surface with no 100nm projections (Fig. 5). *S. aureus* was destroyed after 30 seconds of exposure to nanodroplets at a distance of 5 cm (Fig. 6).

In contrast, for *P. aeruginosa* and *E. coli*, multiple holes with diameters of approximately 100 nm were observed on the bacterial surface after 3 minutes of exposure to nanodroplets at a distance of 17 cm (Fig. 3). For *S. marcescens*, some holes with diameters of approximately 300 nm were observed (Fig. 3). Three Gram-negative bacteria in this state were stained with PI (Fig. 4). However, this tendency was observed only on the surface layer of the biofilm and remained unknown in the underlying layers. Furthermore, the luminance of the calcein-loaded *E. coli* was 59 ± 2 cd/m^2^, whereas it was 41 ± 3 cd/m^2^ after 3 minutes of exposure to nanodroplets at a distance of 17 cm and 31 ± 1 cd/m^2^ after 20 minutes of exposure, decreasing as the exposure time was extended (Fig. S3). Calcein leakage indicated that the nanodroplets penetrated the cell membrane.

At a distance of 5 cm, the bacterial bodies of *P. aeruginosa* were destroyed after 240 seconds exposure and *E. coli* after 90 seconds of exposure (Fig. 6). In *S. marcescens*, after 90 seconds of exposure to nanodroplets, there was a notable decrease in cell counts and the morphology of the cells has altered, displaying what appears to be “holes” or “indents” and some projections after 240 seconds (Fig. 6).

### Compositional analysis of *S. aureus* cell projections after exposure to nanodroplets

**Fig 7:**
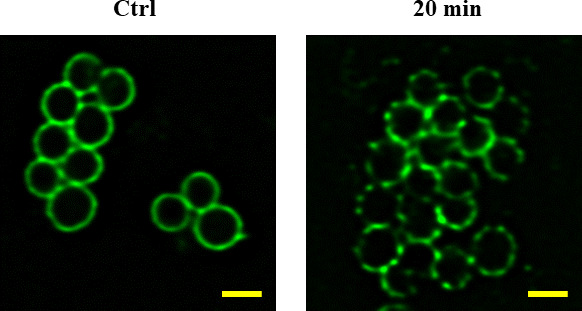
Comparison of fluorescent staining images of the bacterial surface of *S. aureus* with anti-peptidoglycan antibody labeling. *S. aureus*, which had numerous projections on its surface at a distance of 17 cm after exposure to nanodroplets for 20 minutes, was labeled with an anti-peptidoglycan antibody. Ring-shaped fluorescence was observed by CLSM. After imaging with a confocal laser scanning microscope, the images were deconvoluted. The fluorescence intensity is proportional to the labeled quantity of antibody, so regions of high fluorescence intensity indicate high peptidoglycan content. Bar, 1 µm, magnification: ×60.

*S. aureus*, which had numerous projections on its surface after exposure to nanodroplets at a distance of 17 cm for 20 minutes, was labeled with an anti-peptidoglycan antibody. Ring-shaped fluorescence was observed in the cross-section of the bacterial cells using confocal laser scanning microscopy (CLSM) (Fig. 7). The fluorescence intensity of the control was approximately 50,000 a.u. in the overlapping region of the cells and approximately 25,000 a.u. in the non-overlapping region. In contrast, after 20 minutes of exposure, although the fluorescence intensity in the overlapping region was approximately 50,000 a.u., it was 2,000–35,000 a.u. in the other region, and an irregularity was confirmed.

## DISCUSSION

Antimicrobial resistance (AMR) is a major global public health concern. In essence, there is not only the emergence of AMR strains owing to the inappropriate use of antimicrobial agents but also the transmission of these strains through medical staff or facilities. Methicillin-resistant *Staphylococcus aureus* (MRSA) is transmitted to several inpatients through the hands of healthcare workers (27–29). In addition, antibiotic-resistant Gram-negative bacteria, including *P. aeruginosa* and *E. coli*, have been found to colonize drains and sinks in medical facilities, resulting in numerous cases of nosocomial infections originating from these locations (30–34). In some cases, disinfectants were ineffective, and sewage plumbing had to be replaced (31, 34). Nosocomial outbreaks of AMR strains have led to the death of several patients (32, 34, 35). Because effective treatments for drug-resistant bacteria are limited (36), environmental infection control measures are extremely important. Chemical sterilization using disinfectants or physical sterilization using heat or UV radiation are used to prevent contact infection (13, 37, 38). However, some disinfectants harm human health and the environment (39–43). Recently, vaporized hydrogen peroxide, which has a lower environmental impact, has attracted attention. However, disinfection takes several hours (6, 44), during which the area that requires disinfection is inaccessible. Although other studies have shown that UVC reduces the incidence of nosocomial infections and provides economic benefits (45), UVC at a wavelength of 254 nm harms human health (46, 47). UVC at a wavelength of 222 nm used for the sterilization of severe acute respiratory syndrome coronavirus 2 (SARS-CoV-2) is harmless to the eyes or skin (48, 49), and it affects other viruses and planktonic bacteria (4, 50, 51). However, the efficacy against biofilm-forming bacteria has not yet been reported. In this study, we evaluated the bactericidal effect of the world’s first high-speed nanodroplet generation technology on biofilm-forming bacteria. This technology makes a droplet collide with a pathogen at a velocity of up to 50 m/s only using water with an extremely small mass. Since droplets of less than 100 nm in diameter were impacted in this study, the area of propagation was extremely small and only affected the area, and there is no influence on the object. Moreover, when the droplet size falls below 100 nm, the droplet dries instantly due to the Kelvin effect (52), and the contact surface does not become wet.

The nanodroplets generated using this technology have several possible effects when they collide with bacteria. Nanodroplets are extremely small (less than 100 nm) and have a large specific surface area; therefore, an increase in the surface energy of the nanodroplets could be involved in any reaction, while the generation of reactive oxygen species that could cause bactericidal effects has not been confirmed. In addition, because nanodroplets are jetted at high velocities, they can exert a very high impact pressure on the bacteria. In this study, the impact pressure of droplets colliding with the bacterial surface at each distance from the nozzle exit was calculated using the equation below (21).


p=ρ c v,

where *P* is the impact pressure, *ρ* is the liquid density, *c* is the speed of sound of the liquid, and *v* is the droplet velocity. Furthermore, we have confirmed that the theoretical value of impact pressure is on the same order of magnitude as the actual measurement. The droplet velocities *v* were 12 m/s at 17 cm and 50 m/s at 5 cm. For water, *ρ* = 1,000 kg/m^3^ and *c* = 1,500 m/s; the impact pressure is 1.8 × 10^7^ Pa (18 MPa) at 17 cm and 7.5 × 10^7^ Pa (75 MPa) at 5 cm. The shorter the distance from the nozzle, the greater the droplet velocity, which allows for a stronger impact at a closer distance. As the size decreases, the Laplace pressure (internal pressure) of the nanodroplets increases; therefore, there is a possibility that the nanodroplets can penetrate bacterial cells without breaking after collision at the calculated impact pressure. Although the range of droplet size is not known, since the larger the droplet, the greater its momentum, it can be assumed that larger droplets will damage the cell wall due to impact pressure and push-in caused by high-velocity impact. On the other hand, as the droplet size decreases, the extent and depth of damage is expected to decrease; however, the smaller droplet size may well cause damage because the number of droplets is extremely large. Although we have not yet elucidated the mechanism of damage depending on droplet size, this is an important issue that we plan to address in future research.

In this study, after 30 seconds of exposure to nanodroplets at a distance of 5 cm (impact pressure: 75 MPa), *S. aureus* on the biofilm model was completely sterilized, although approximately 1/10,000th of the viable bacteria were dispersed. At this time, the bacterial cells on the biofilm model were completely destroyed. Therefore, we considered that the high-speed collision of nanodroplets triggered an explosion of the bacterial body with a turgor pressure of ~20 atm (53). This novel mechanism differs from chemical reactions with bacterial cell membranes and enzymes or nucleic acid mutations caused by disinfection techniques, including various disinfectants and UVC.

However, at a distance of 17 cm (impact pressure: 18 MPa), *S. aureus* was not sterilized after 20 minutes of exposure to nanodroplets. At this point, the bacterial cells were not stained with PI, which binds to DNA. If the cell membrane is damaged, it enters the nucleus and binds to DNA, causing it to fluoresce (54). In addition, the luminance of the calcein did not change. Calcein-AM passes through the cell membrane of viable bacteria and is hydrolyzed by esterases to membrane-impermeable calcein, which is retained within the cell (55). Since calcein fluorescence leaks out when cells suffer membrane damage (55, 56), the effect of nanodroplets can be determined by fluorescence leakage, so no calcein leakage was observed. In other words, the nanodroplets did not reach the cell membrane at an impact pressure of 18 MPa. Numerous projections were observed on the surface of *S. aureus*. Because the cell membrane was not damaged, we hypothesized that the projection component was peptidoglycan, which resides in the upper layer above the cell membrane. Therefore, we observed the distribution of peptidoglycans on the bacterial surface by CLSM using an anti-peptidoglycan antibody that specifically labels peptidoglycans. This microscope focuses the laser beam on the confocal plane to obtain a cross-sectional image of the object so that only the outer side of the bacteria was stained with fluorescence, like a ring (Fig. 7). Because the quantity of antibody labeling was proportional to that of peptidoglycan, the fluorescence intensity was higher in the overlapping regions of the bacteria. The nanodroplets were not expected to hit the overlapping region; therefore, no change in the fluorescence intensity was observed even after 20 minutes of exposure. In contrast, the fluorescence intensity was non-uniform in the non-overlapping regions of the cells, and the outer side of the cell was observed as a dotted line. At this time, the fluorescence intensity of the point-like region was higher than that of the control. A difference in the quantity labeled by the anti-peptidoglycan antibody may have occurred in the projection and other regions of the peptidoglycan that had been pushed outside the bacterial body through a lesion by the turgor pressure. Therefore, we concluded that the projection components that emerged on the bacterial body surface were peptidoglycans. In addition, Gram staining confirmed that *S. aureus* strains of the state mentioned above were pink (data not shown). Therefore, crystal violet staining was thought to be easily decolorized by the collapse of the mesh-like framework structure in the peptidoglycan layer. This was another result supporting damage to the peptidoglycan layer. When *S. aureus* in this state was incubated on MHA for 24 hours, colonies formed again, and it was confirmed by scanning electron microscopy (SEM) that these returned to the normal bacterial surface. It was newly found that *S. aureus* can survive and repair peptidoglycan projections from bacterial surfaces.

*S. aureus* could continue living while maintaining its bacterial form even though an impact pressure of 18 MPa damaged the cell wall. However, the bacterial bodies exploded completely at an impact pressure of 75 MPa. At this stage, it was suggested that the velocity at which the nanodroplets collided with the bacterial surface affected the bactericidal effect. However, a droplet is water vapor until it is jetted from the nozzle, after which it becomes a droplet due to condensation. The droplet size increases as it moves downstream and decreases when evaporation exceeds condensation. Therefore, when the distance from a nozzle becomes 5 cm, it is considered that the droplet diameter should be smaller. Further examination of the droplet size and impact pressure is required.

Although the three Gram-negative bacteria were not completely sterilized at a distance of 17 cm (impact pressure: 18 MPa), multiple holes were observed on each bacterial surface. The holes formed on the bacterial surface of *S. marcescens* were larger than those on the surfaces of *P. aeruginosa* and *E. coli*. Furthermore, when LIVE/DEAD staining was conducted over time, *S. marcescens* took the longest until the cells in the entire observation field were stained with PI (data not shown). *Serratia* spp. produce lipopeptides, such as serrawettins and stephemsiolides (57–60). These are biosurfactants that reduce the surface tension of water (57–59). Thus, in *S. marcescens*, the power of the high-speed nanodroplets was probably reduced because the droplet size that hit the bacterial surface might be increased instantaneously as the surface tension of the nanodroplets decreased. Damage to the cell membranes of the three Gram-negative bacteria, including *S. marcescens*, was observed at a distance of 17 cm, indicating that the cell wall could not withstand an impact pressure of 18 MPa, and these bacteria were more fragile than *S. aureus* with projections formed on the bacterial surface, but not stained by PI. Furthermore, the luminance of calcein-loaded *E. coli* decreased. When the cell membrane is damaged by the nanodroplets, calcein leaks out of there, causing a decrease in luminance. Therefore, the absence of calcein leakage in *S. aureus*, while it was observed in *E. coli*, also suggested cell wall fragility. However, *P. aeruginosa* and *S. marcescens* did not stain well with calcein in the first place. This was a limitation of this study. Despite the fragile cell wall of Gram-negative bacteria, the viable count of biofilm-forming bacteria was not significantly reduced in this study. This is because the nanodroplets may not have reliably affected all bacteria at a distance of 17 cm.

However, at a distance of 5 cm (impact pressure: 75 MPa), *E. coli* and *S. marcescens* after 90 seconds of exposure and *P. aeruginosa* after 240 seconds of exposure were physically destroyed and completely sterilized. At this time, only approximately 1/10,000th of the surviving bacteria were dispersed around the biofilm model. Even among the Gram-negative bacteria, the biofilm biomass of *P. aeruginosa* was more than six times greater than that of *E. coli* and *S. marcescens*. In addition, planktonic cells were sterilized in a shorter time than the biofilm-forming bacteria. Therefore, biofilm formation was considered to affect the bactericidal effects by nanodroplets, and it suggested that biofilm-forming *P. aeruginosa* required the longest time to be completely sterilized.

In summary, nanodroplets with an impact of 75 MPa killed all four bacterial species used in this study. Nanodroplets with an impact pressure of 18 MPa damaged the cell walls of *S. aureus* and extruded peptidoglycans, which were repaired and survived. In contrast, Gram-negative bacteria were sterilized by the nanodroplets, but the sterilization ability depended on the biofilm biomass.

This technology is an effective disinfection method for both Gram-positive and Gram-negative bacteria. Furthermore, because this disinfection method uses only water, we believe it will help solve the problems of human toxicity and environmental pollution caused by existing disinfection methods. Currently, the exposure range is narrow, so scaling up for practical use is a major challenge, but we would like to consider multiple methods in the future.

## MATERIALS AND METHODS

### Bacterial strains and *in vitro* biofilm models

The bacterial strains used in this study were *S. aureus* ATCC29213, *P. aeruginosa* PAO1, *E. coli* ATCC25922, and a clinical isolate of *S. marcescens*. *In vitro* biofilm models of these four bacterial species were prepared on a stainless disk, Φ10 mm, contact lens, and cover glass, Φ15 mm. Various bacteria were cultured on MHA (Eiken Chemical) at 35°C for 24 hours, and 100 µL of this bacterial solution adjusted to McFarland No. 0.5 was mixed with 10 mL of tryptic soy broth (TSB: Becton, Dickinson and Company). Sterile stainless disks were immersed in the culture medium and incubated at 35°C for 24 hours with gentle shaking (61). In addition, a cover glass and contact lens washed with phosphate-buffered saline (PBS: Fujifilm Wako Pure Chemical) were placed on the agar (300 µL) in a Petri dish (Φ90 mm), respectively. Two hundred microliters of each bacterial solution was added on the above-mentioned cover glass or contact lens and cultured at 35°C for 24 hours with gentle shaking (61). Biofilm formation on these material surfaces was observed using a scanning electron microscope VE-8800 (SEM: Keyence).

### Quantification of biofilm biomass

Quantification of the biofilm biomass of the four bacterial species was performed using a crystal violet assay (62). Biofilm-formed contact lens models were washed using PBS after the removal of the culture medium. Two hundred microliters of 0.1% crystal violet was added and incubated at 25°C for 20 minutes. The crystal violet was subsequently removed, and the biofilm was washed with PBS. Each biofilm formation model was immersed in 1 mL of 99.5% ethanol for 30 minutes, and the absorbance (595 nm) of the dissolved crystal violet was measured using an iMark microplate reader (Bio-Rad).

### High-speed nanodroplet-generating device

High-speed nanodroplet-generating device which was developed in 2017 was used in this study (Fig. 1) (20). This technology has the ability to gush out water nanodroplets of less than 100 nm at a velocity of ~50 m/s and a temperature of ~30°C at a distance of 5 cm from the nozzle exit without wetting the contact surface. When the device was placed on a desk, the distance from the nozzle tip to desk was 17 cm (Fig. 1); therefore, the exposure distance was set to 17 cm or 5 cm from the nozzle. When the biofilm models were exposed to the nanodroplets, the room temperature was 20.1°C–27.5°C. In addition, relative humidity affects the diameter of the droplet produced (63). However, since experiments were conducted in the range of 20.0%–31.0% relative humidity, droplet diameters were assumed to be generally the same, and therefore, the effect of relative humidity was negligible. The maximum operating time of the device was 25 minutes.

### Viable count after exposure to nanodroplets

For each biofilm-formed contact lens model of the four bacterial species, the nanodroplets were exposed for 15, 30, 45, 60, 90, 120, 180, and 1,200 seconds (20 minutes) at a distance of 17 cm and for 15, 30, 45, 90, 120, 180, and 240 seconds at a distance of 5 cm. Each exposed model surface was swabbed at any of the three locations, suspended in 1 mL PBS, and inoculated onto agar. After incubation at 35°C for 24 hours, the viable counts were measured. Similar experiments were also performed with planktonic bacteria. In addition, the viable bacteria dispersed around the biofilm model after exposure to nanodroplets was also counted. Each biofilm-formed contact lens model of four bacterial species was surrounded by MHB, the nanodroplets were exposed for 1,200 seconds (20 minutes) at a distance of 17 cm, and for each bacteria was completely sterilized time (*S. aureus*: 30 seconds, *P. aeruginosa*: 240 seconds, *E. coli*: 90 seconds, and *S. marcescens*: 90 seconds) at a distance of 5 cm. After incubation at 35°C for 24 hours, the viable counts of around the biofilm model were measured.

### Mechanism of sterilization by exposure to nanodroplets

A stainless disk was selected as the sample for the SEM to observe the morphology of the bacteria. Biofilm models of the four bacterial species were exposed to nanodroplets at a distance of 17 cm for 3 and 20 minutes and at 5 cm for 30, 90, and 240 seconds. Morphological changes were observed by SEM after fixation and dehydration. For SEM pretreatment, the samples were fixed with 2.5% glutaraldehyde for 30 minutes and then dehydrated with 50%, 75%, and 99.5% ethanol for 5 minutes at each step. After coating with gold, the samples were observed at an accelerating voltage of 8 kV (64).

In addition, biofilm models of the four bacterial species were prepared using a contact lens capable of transmitting excitation light from a fluorescence microscope. These models were then stained with the LIVE/DEAD BacLight Bacterial Viability Kit (Thermo Fisher Scientific). That is, 20 µL of the reagent, prepared according to the attached document, was added onto the sample and incubated in the dark for 20 minutes. The samples were observed under a fluorescence microscope BZ-X800 (Keyence).

The effect of the nanodroplets on the cells was further examined by loading the cells with calcein (55, 56). *S. aureus* and *E. coli* were used in this study. A 50µL aliquot of calcein-AM (10 µg/mL, Fujifilm Wako Pure Chemical) was added to 100 µL of the bacterial solution at the same concentration as used in the biofilm model preparation. After this solution was added to the cover glass, the cells were incubated at 35°C for 24 hours and then washed once with PBS. Calcein-loaded *S. aureus* and *E. coli* were exposed to nanodroplets at a distance of 17 cm for 3 and 20 minutes, respectively, and then observed using a fluorescence microscope BZ-X800 with identical exposure times. The luminance of the images was measured.

### Changes in the surface of *S. aureus* and elucidation of its composition after exposure to nanodroplets

The laser beam-transmitting material of the confocal laser scanning microscope was used, and the biofilm-formed cover glass model was chosen because of the nonspecific reaction of the contact lens with the antibody. The biofilm-forming *S. aureus* cells were exposed to nanodroplets at a distance of 17 cm for 20 minutes and then stained using the method described by Schweitzer *et al.* (65) and Ouchi (66). The samples were fixed in 99.8% methanol at −20°C for 5 minutes and then immersed in 0.5% Triton X-100 (Sigma-Aldrich) for 20 minutes at 25°C. After washing three times with PBS, the samples were incubated with 100 µL of 2% skim milk for 30 minutes at 25°C. Subsequently, mouse anti-peptidoglycan 7263–1006 (Bio-Rad) was diluted at a ratio of 1:75 with 2% skim milk in PBS, and each sample was incubated at 37°C for 90 minutes after dropping 100 µL of it. After incubation, the samples were washed three times with PBS, and 100 µL of FITC-conjugated AffiniPure goat anti-mouse IgG (H + L) SA00003-1 (Proteintech), which was diluted at a ratio of 1:500 with 2% skim milk in PBS, was added; they were then incubated at 37°C for 90 minutes. The samples were washed thrice with PBS and mounted in ImmunoSelect Antifading Mounting Medium (Dionova). The stained samples were observed using a confocal laser scanning microscope FLUOVIEW FV3000 (CLSM: Olympus). Observations were made using an Alexa Fluor 488 laser at an excitation wavelength of 495 nm and a fluorescence wavelength of 519 nm at an intensity of 0.01%.

## SUPPLEMENTAL MATERIAL

Figure S1 jb.00139-24-s0001.tiff Changes in viable count over time of each bacterial planktonic cell.

Figure S2 jb.00139-24-s0002.tiff SEM images of the bacterial surface of *S. aureus*.

Figure S3 jb.00139-24-s0003.tiff Effect of the nanodroplets on *S. aureus* and *E. coli* cells containing calcein.

ASM does not own the copyrights to Supplemental Material that may be linked to, or accessed through, an article. The authors have granted ASM a non-exclusive, world-wide license to publish the Supplemental Material files. Please contact the corresponding author directly for reuse.

## References

[1] Lederberg J. 2000. Infectious history. Science 288:287–293. doi:10.1126/science.288.5464.287 10777411

[2] Landecker H. 2019. Antimicrobials before antibiotics: war, peace, and disinfectants. Palgrave Commun 5:45. doi:10.1057/s41599-019-0251-8

[3] Hockberger PE. 2002. A history of ultraviolet photobiology for humans, animals and microorganisms. Photochem Photobiol 76:561–579. doi:10.1562/0031-8655(2002)0760561AHOUPF2.0.CO2 12511035

[4] Narita K, Asano K, Naito K, Ohashi H, Sasaki M, Morimoto Y, Igarashi T, Nakane A. 2020. Ultraviolet C light with wavelength of 222 nm inactivates a wide spectrum of microbial pathogens. J Hosp Infect 105:459–467. doi:10.1016/j.jhin.2020.03.030 32243946

[5] Doidge M, Allworth AM, Woods M, Marshall P, Terry M, O’Brien K, Goh HM, George N, Nimmo GR, Schembri MA, Lipman J, Paterson DL. 2010. Control of an outbreak of carbapenem-resistant Acinetobacter baumannii in Australia after introduction of environmental cleaning with a commercial oxidizing disinfectant. Infect Control Hosp Epidemiol 31:418–420. doi:10.1086/651312 20175684

[6] Totaro M, Casini B, Profeti S, Tuvo B, Privitera G, Baggiani A. 2020. Role of hydrogen peroxide vapor (HPV) for the disinfection of hospital surfaces contaminated by multiresistant bacteria. Pathogens 9:408. doi:10.3390/pathogens9050408 32456303 PMC7281489

[7] Lin W, Guan X, Cao J, Niu B, Chen Q. 2017. Bactericidal mechanism of glutaraldehyde-didecyldimethylammonium bromide as a disinfectant against Escherichia coli. J Appl Microbiol 122:676–685. doi:10.1111/jam.13384 27997750

[8] Kim JY, Lee C, Cho M, Yoon J. 2008. Enhanced inactivation of E. coli and MS-2 phage by silver ions combined with UV-A and visible light irradiation. Water Res 42:356–362. doi:10.1016/j.watres.2007.07.024 17692890

[9] Elasri MO, Miller RV. 1999. Study of the response of a biofilm bacterial community to UV radiation. Appl Environ Microbiol 65:2025–2031. doi:10.1128/AEM.65.5.2025-2031.1999 10223995 PMC91292

[10] Römling U, Balsalobre C. 2012. Biofilm infections, their resilience to therapy and innovative treatment strategies. J Intern Med 272:541–561. doi:10.1111/joim.12004 23025745

[11] Slipski CJ, Jamieson TR, Zhanel GG, Bay DC. 2020. Riboswitch-associated guanidinium-selective efflux pumps frequently transmitted on proteobacterial plasmids increase Escherichia coli biofilm tolerance to disinfectants. J Bacteriol 202:e00104-20. doi:10.1128/JB.00104-20 32928929 PMC7648145

[12] Luo X, Zhang B, Lu Y, Mei Y, Shen L. 2022. Advances in application of ultraviolet irradiation for biofilm control in water and wastewater infrastructure. J Hazard Mater 421:126682. doi:10.1016/j.jhazmat.2021.126682 34388918

[13] Maillard JY, Centeleghe I. 2023. How biofilm changes our understanding of cleaning and disinfection. Antimicrob Resist Infect Control 12:95. doi:10.1186/s13756-023-01290-4 37679831 PMC10483709

[14] Gaze WH, Abdouslam N, Hawkey PM, Wellington EMH. 2005. Incidence of class 1 integrons in a quaternary ammonium compound-polluted environment. Antimicrob Agents Chemother 49:1802–1807. doi:10.1128/AAC.49.5.1802-1807.2005 15855499 PMC1087628

[15] Mc Cay PH, Ocampo-Sosa AA, Fleming GTA. 2010. Effect of subinhibitory concentrations of benzalkonium chloride on the competitiveness of Pseudomonas aeruginosa grown in continuous culture. Microbiol (Reading) 156:30–38. doi:10.1099/mic.0.029751-0 19815578

[16] Ghorbal SKB, Chourabi K, Maalej L, Ammar AB, Ouzari HI, Hassen A, Jaafoura H, Chatti A. 2019. Pseudomonas aeruginosa swarmer cells adaptation toward UVc radiations. Front Microbiol 10:556. doi:10.3389/fmicb.2019.00556 31001210 PMC6454200

[17] Jaiaue P, Piluk J, Sawattrakool K, Thammakes J, Malasuk C, Thitiprasert S, Thongchul N, Siwamogsatham S. 2022. Mathematical modeling for evaluating inherent parameters affecting UVC decontamination of indicator bacteria. Appl Environ Microbiol 88:e0214821. doi:10.1128/aem.02148-21 35289640 PMC9004376

[18] Dulay MT, Lee JK, Mody AC, Narasimhan R, Monack DM, Zare RN. 2020. Spraying small water droplets acts as a bacteriocide. QRB Discov 1:e3. doi:10.1017/qrd.2020.2 37528962 PMC10392691

[19] Lee JK, Walker KL, Han HS, Kang J, Prinz FB, Waymouth RM, Nam HG, Zare RN. 2019. Spontaneous generation of hydrogen peroxide from aqueous microdroplets. Proc Natl Acad Sci U S A 116:19294–19298. doi:10.1073/pnas.1911883116 31451646 PMC6765303

[20] Xiao Y, Liu S, Nakajima T, Sato T. 2022. Characteristics of high-speed mist generated by condensation of water vapor in pressurized air. Int J Plasma Environ Sci Technol 16:e03003. doi:10.34343/ijpest.2022.16.e03003

[21] Uehara S, Nakajima T, Moriya S, Maruyama S, Sato T. 2019. Removal mechanism of artificial dental plaque by impact of micro-droplets. ECS J Solid State Sci Technol 8:20–N24. doi:10.1149/2.0091902jss

[22] Liao X, Xia X, Yang H, Zhu Y, Deng R, Ding T. 2023. Bacterial drug-resistance and viability phenotyping upon disinfectant exposure revealed by single-nucleotide resolved-allele specific isothermal RNA amplification. J Hazard Mater 448:130800. doi:10.1016/j.jhazmat.2023.130800 36716555 PMC9883656

[23] Rackur H. 1985. New aspects of mechanism of action of povidone-iodine. J Hosp Infect 6:13–23. doi:10.1016/s0195-6701(85)80041-4 2860158

[24] Linley E, Denyer SP, McDonnell G, Simons C, Maillard JY. 2012. Use of hydrogen peroxide as a biocide: new consideration of its mechanisms of biocidal action. J Antimicrob Chemother 67:1589–1596. doi:10.1093/jac/dks129 22532463

[25] Rosenberg B, Kemeny G, Switzer RC, Hamilton TC. 1971. Quantitative evidence for protein denaturation as the cause of thermal death. Nature 232:471–473. doi:10.1038/232471a0 4937206

[26] Qiu X, Sundin GW, Wu L, Zhou J, Tiedje JM. 2005. Comparative analysis of differentially expressed genes in Shewanella oneidensis MR-1 following exposure to UVC, UVB, and UVA radiation. J Bacteriol 187:3556–3564. doi:10.1128/JB.187.10.3556-3564.2005 15866945 PMC1111996

[27] Ho SSK, Tse MMY, Boost MV. 2012. Effect of an infection control programme on bacterial contamination of enteral feed in nursing homes. J Hosp Infect 82:49–55. doi:10.1016/j.jhin.2012.05.002 22765960

[28] Hocine MN, Temime L. 2015. Impact of hand hygiene on the infectious risk in nursing home residents: a systematic review. Am J Infect Control 43:e47–e52. doi:10.1016/j.ajic.2015.05.043 26184767

[29] Brown NM, Reacher M, Rice W, Roddick I, Reeve L, Verlander NQ, Broster S, Ogilvy-Stuart AL, D’Amore A, Ahluwalia J, Robinson S, Thaxter R, Moody C, Kearns A, Greatorex J, Martin H, Török ME, Enoch DA. 2019. An outbreak of meticillin-resistant Staphylococcus aureus colonization in a neonatal intensive care unit: use of a case-control study to investigate and control it and lessons learnt. J Hosp Infect 103:35–43. doi:10.1016/j.jhin.2019.05.009 31132394

[30] De Geyter D, Blommaert L, Verbraeken N, Sevenois M, Huyghens L, Martini H, Covens L, Piérard D, Wybo I. 2017. The sink as a potential source of transmission of carbapenemase-producing Enterobacteriaceae in the intensive care unit. Antimicrob Resist Infect Control 6:24. doi:10.1186/s13756-017-0182-3 28239453 PMC5314675

[31] Decraene V, Phan HTT, George R, Wyllie DH, Akinremi O, Aiken Z, Cleary P, Dodgson A, Pankhurst L, Crook DW, Lenney C, Walker AS, Woodford N, Sebra R, Fath-Ordoubadi F, Mathers AJ, Seale AC, Guiver M, McEwan A, Watts V, Welfare W, Stoesser N, Cawthorne J, TRACE Investigators’ Group. 2018. A large, refractory nosocomial outbreak of Klebsiella pneumoniae carbapenemase-producing Escherichia coli demonstrates carbapenemase gene outbreaks involving sink sites require novel approaches to infection control. Antimicrob Agents Chemother 62:e01689-18. doi:10.1128/AAC.01689-18 30249685 PMC6256751

[32] Catho G, Martischang R, Boroli F, Chraïti MN, Martin Y, Koyluk Tomsuk Z, Renzi G, Schrenzel J, Pugin J, Nordmann P, Blanc DS, Harbarth S. 2021. Outbreak of Pseudomonas aeruginosa producing VIM carbapenemase in an intensive care unit and its termination by implementation of waterless patient care. Crit Care 25:301. doi:10.1186/s13054-021-03726-y 34412676 PMC8376114

[33] Brehony C, Domegan L, Foley M, Fitzpatrick M, Cafferkey JP, O’Connell K, Dinesh B, McNamara E, Duffy F, Fitzpatrick F, Burns K. 2021. Molecular epidemiology of an extended multiple-species OXA-48 CPE outbreak in a hospital ward in Ireland, 2018–2019. Antimicrob Steward Healthc Epidemiol 1:e54. doi:10.1017/ash.2021.206 36168481 PMC9495434

[34] Schärer V, Meier MT, Schuepbach RA, Zinkernagel AS, Boumasmoud M, Chakrakodi B, Brugger SD, Fröhlich MR, Wolfensberger A, Sax H, Kuster SP, Schreiber PW. 2023. An intensive care unit outbreak with multi-drug-resistant Pseudomonas aeruginosa – spotlight on sinks. J Hosp Infect 139:161–167. doi:10.1016/j.jhin.2023.06.013 37343769

[35] Jiang A, Shi X, Zheng H, Liu N, Chen S, Gao H, Ren M, Zheng X, Fu X, Liang X, Ruan Z, Tian T, Yao Y. 2022. Establishment and validation of a nomogram to predict the in-hospital death risk of nosocomial infections in cancer patients. Antimicrob Resist Infect Control 11:29. doi:10.1186/s13756-022-01073-3 35130978 PMC8822816

[36] Chng KR, Li C, Bertrand D, Ng AHQ, Kwah JS, Low HM, Tong C, Natrajan M, Zhang MH, Xu L, Ko KKK, Ho EXP, Av-Shalom TV, Teo JWP, Khor CC, Consortium M, Chen SL, Mason CE, Ng OT, Marimuthu K, Ang B, Nagarajan N. 2020. Cartography of opportunistic pathogens and antibiotic resistance genes in a tertiary hospital environment. Nat Med 26:941–951. doi:10.1038/s41591-020-0894-4 32514171 PMC7303012

[37] Boyce JM. 2016. Modern technologies for improving cleaning and disinfection of environmental surfaces in hospitals. Antimicrob Resist Infect Control 5:10. doi:10.1186/s13756-016-0111-x 27069623 PMC4827199

[38] Kizny Gordon AE, Mathers AJ, Cheong EYL, Gottlieb T, Kotay S, Walker AS, Peto TEA, Crook DW, Stoesser N. 2017. The hospital water environment as a reservoir for carbapenem-resistant organisms causing hospital-acquired infections-a systematic review of the literature. Clin Infect Dis 64:1435–1444. doi:10.1093/cid/cix132 28200000

[39] Jing JLJ, Pei Yi T, Bose RJC, McCarthy JR, Tharmalingam N, Madheswaran T. 2020. Hand sanitizers: a review on formulation aspects, adverse effects, and regulations. Int J Environ Res Public Health 17:3326. doi:10.3390/ijerph17093326 32403261 PMC7246736

[40] Wang Y, Wu Q, Ren B, Muskhelishvili L, Davis K, Wynne R, Rua D, Cao X. 2022. Subacute pulmonary toxicity of glutaraldehyde aerosols in a human in vitro airway tissue model. Int J Mol Sci 23:12118. doi:10.3390/ijms232012118 36292975 PMC9603730

[41] Mohapatra S, Yutao L, Goh SG, Ng C, Luhua Y, Tran NH, Gin K-H. 2023. Quaternary ammonium compounds of emerging concern: classification, occurrence, fate, toxicity and antimicrobial resistance. J Hazard Mater 445:130393. doi:10.1016/j.jhazmat.2022.130393 36455328 PMC9663149

[42] Kameda T, Oka S, Igawa JI, Sakamoto M, Terada K. 2022. Can hypochlorous acid be a powerful sanitizer to replace alcohol for disinfection? -Its bactericidal, degradation of the solutions under various storage condition, and steel rust effects. Dent Mater J 41:167–183. doi:10.4012/dmj.2021-146 34690228

[43] He H, Li F, Liu K, Zhan J, Wang X, Lai C, Yang X, Huang B, Pan X. 2023. The disinfectant residues promote the leaching of water contaminants from plastic pipe particles. Environ Pollut 327:121577. doi:10.1016/j.envpol.2023.121577 37023886

[44] Otter JA, French GL. 2009. Survival of nosocomial bacteria and spores on surfaces and inactivation by hydrogen peroxide vapor. J Clin Microbiol 47:205–207. doi:10.1128/JCM.02004-08 18971364 PMC2620839

[45] Raggi R, Archulet K, Haag CW, Tang W. 2018. Clinical, operational, and financial impact of an ultraviolet-C terminal disinfection intervention at a community hospital. Am J Infect Control 46:1224–1229. doi:10.1016/j.ajic.2018.05.012 29934205

[46] Delic NC, Lyons JG, Di Girolamo N, Halliday GM. 2017. Damaging effects of ultraviolet radiation on the cornea. Photochem Photobiol 93:920–929. doi:10.1111/php.12686 27935054

[47] Welch D, Buonanno M, Grilj V, Shuryak I, Crickmore C, Bigelow AW, Randers-Pehrson G, Johnson GW, Brenner DJ. 2018. Far-UVC light: a new tool to control the spread of airborne-mediated microbial diseases. Sci Rep 8:2752. doi:10.1038/s41598-018-21058-w 29426899 PMC5807439

[48] Kaidzu S, Sugihara K, Sasaki M, Nishiaki A, Igarashi T, Tanito M. 2019. Evaluation of acute corneal damage induced by 222-nm and 254-nm ultraviolet light in Sprague-Dawley rats. Free Radic Res 53:611–617. doi:10.1080/10715762.2019.1603378 30947566

[49] Yamano N, Kunisada M, Kaidzu S, Sugihara K, Nishiaki-Sawada A, Ohashi H, Yoshioka A, Igarashi T, Ohira A, Tanito M, Nishigori C. 2020. Long-term effects of 222-nm ultraviolet radiation C sterilizing lamps on mice susceptible to ultraviolet radiation. Photochem Photobiol 96:853–862. doi:10.1111/php.13269 32222977 PMC7497027

[50] Kitagawa H, Nomura T, Nazmul T, Omori K, Shigemoto N, Sakaguchi T, Ohge H. 2021. Effectiveness of 222-nm ultraviolet light on disinfecting SARS-CoV-2 surface contamination. Am J Infect Control 49:299–301. doi:10.1016/j.ajic.2020.08.022 32896604 PMC7473342

[51] Chen H, Moraru CI. 2023. Exposure to 222 nm far UV-C effectively inactivates planktonic foodborne pathogens and inhibits biofilm formation. Innov Food Sci Emerg Technol 87:103411. doi:10.1016/j.ifset.2023.103411

[52] Ang EYM, Wang PC, Toh W, Ng TY. 2023. Suspended water nanodroplets evaporation and its deviation from continuum estimations. J Mol Liq 370:121034. doi:10.1016/j.molliq.2022.121034

[53] Erickson HP. 2021. How teichoic acids could support a periplasm in Gram-positive bacteria, and let cell division cheat turgor pressure. Front Microbiol 12:664704. doi:10.3389/fmicb.2021.664704 34040598 PMC8141598

[54] Berney M, Hammes F, Bosshard F, Weilenmann HU, Egli T. 2007. Assessment and interpretation of bacterial viability by using the LIVE/DEAD BacLight Kit in combination with flow cytometry. Appl Environ Microbiol 73:3283–3290. doi:10.1128/AEM.02750-06 17384309 PMC1907116

[55] Takenaka S, Trivedi HM, Corbin A, Pitts B, Stewart PS. 2008. Direct visualization of spatial and temporal patterns of antimicrobial action within model oral biofilms. Appl Environ Microbiol 74:1869–1875. doi:10.1128/AEM.02218-07 18223108 PMC2268315

[56] Hossain F, Dohra H, Yamazaki M. 2021. Effect of membrane potential on entry of lactoferricin B-derived 6-residue antimicrobial peptide into single Escherichia coli cells and lipid vesicles. J Bacteriol 203:e00021-21. doi:10.1128/JB.00021-21 33558393 PMC8092161

[57] Matsuyama T, Kaneda K, Nakagawa Y, Isa K, Hara-Hotta H, Yano I. 1992. A novel extracellular cyclic lipopeptide which promotes flagellum-dependent and -independent spreading growth of Serratia marcescens. J Bacteriol 174:1769–1776. doi:10.1128/jb.174.6.1769-1776.1992 1548227 PMC205777

[58] Clements T, Ndlovu T, Khan S, Khan W. 2019. Biosurfactants produced by Serratia species: classification, biosynthesis, production and application. Appl Microbiol Biotechnol 103:589–602. doi:10.1007/s00253-018-9520-5 30456577

[59] Zhang K, Tao W, Lin J, Wang W, Li S. 2021. Production of the biosurfactant serrawettin W1 by Serratia marcescens S-1 improves hydrocarbon degradation. Bioprocess Biosyst Eng 44:2541–2552. doi:10.1007/s00449-021-02625-4 34514513

[60] Clements-Decker T, Rautenbach M, Khan S, Khan W. 2022. Metabolomics and genomics approach for the discovery of serrawettin W2 lipopeptides from Serratia marcescens NP2. J Nat Prod 85:1256–1266. doi:10.1021/acs.jnatprod.1c01186 35438991

[61] Gu H, Lee SW, Carnicelli J, Jiang Z, Ren D. 2019. Antibiotic susceptibility of Escherichia coli cells during early-stage biofilm formation. J Bacteriol 201:e00034-19. doi:10.1128/JB.00034-19 31061169 PMC6707912

[62] Akinbobola AB, Sherry L, Mckay WG, Ramage G, Williams C. 2017. Tolerance of Pseudomonas aeruginosa in in-vitro biofilms to high-level peracetic acid disinfection. J Hosp Infect 97:162–168. doi:10.1016/j.jhin.2017.06.024 28648453

[63] Dulay MT, Chamberlayne CF, Zare RN. 2022. Optimizing coaxial sonic spray geometry for generating water microdroplets. Anal Chem 94:3762–3766. doi:10.1021/acs.analchem.1c05337 35191692

[64] Fujimura S, Sato T, Mikami T, Kikuchi T, Gomi K, Watanabe A. 2008. Combined efficacy of clarithromycin plus cefazolin or vancomycin against Staphylococcus aureus biofilms formed on titanium medical devices. Int J Antimicrob Agents 32:481–484. doi:10.1016/j.ijantimicag.2008.06.030 18790609

[65] Schweitzer MH, Moyer AE, Zheng W. 2016. Testing the hypothesis of biofilm as a source for soft tissue and cell-like structures preserved in dinosaur bone. PLoS One 11:e0150238. doi:10.1371/journal.pone.0150238 26926069 PMC4771714

[66] Ochi T. 2002. Role of mitotic motors, dynein and kinesin, in the induction of abnormal centrosome integrity and multipolar spindles in cultured V79 cells exposed to dimethylarsinic acid. Mutat Res 499:73–84. doi:10.1016/s0027-5107(01)00266-4 11804606

