# Supplementary figures and images for: Impact of high-speed nanodroplets on various pathogenic bacterial cell walls
Source: J Bacteriol. 2024 Oct 9;206(11):e00139-24. doi: 10.1128/jb.00139-24 (PMC11580407; doi:10.1128/jb.00139-24)

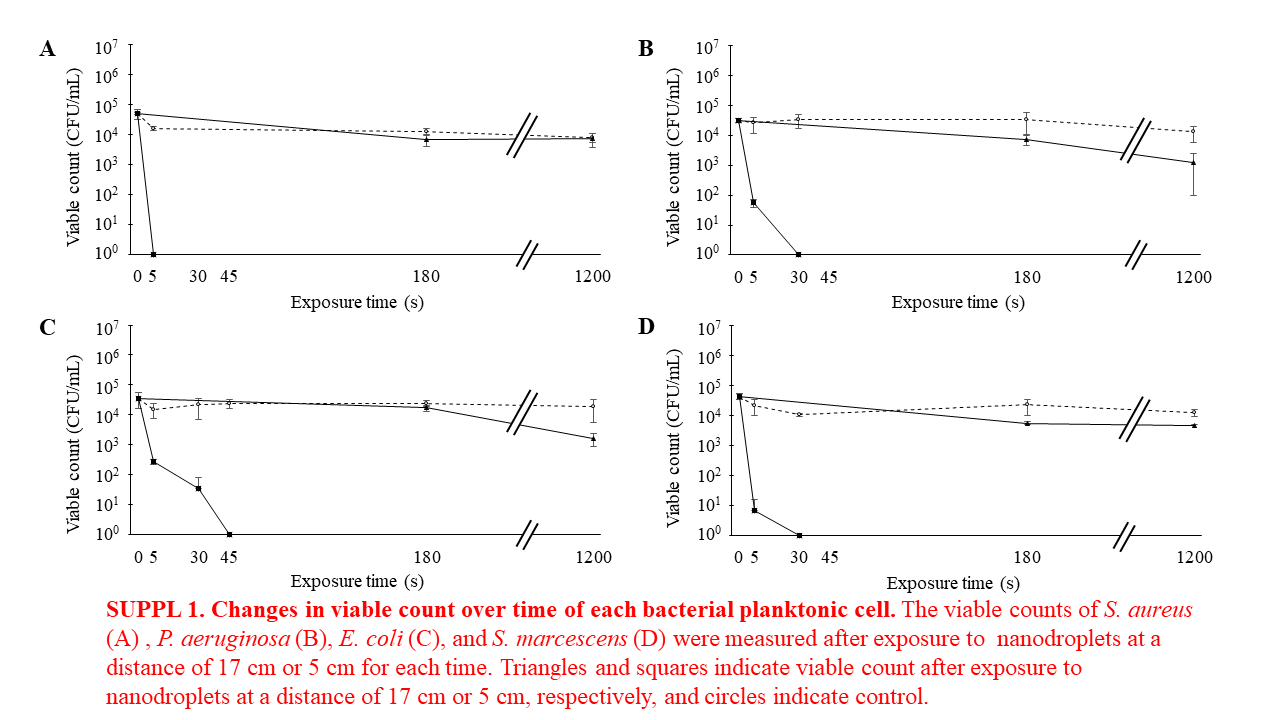

Supplement: Figure S1 — Changes in viable count over time of each bacterial planktonic cell. [file jb.00139-24-s0001.tiff]

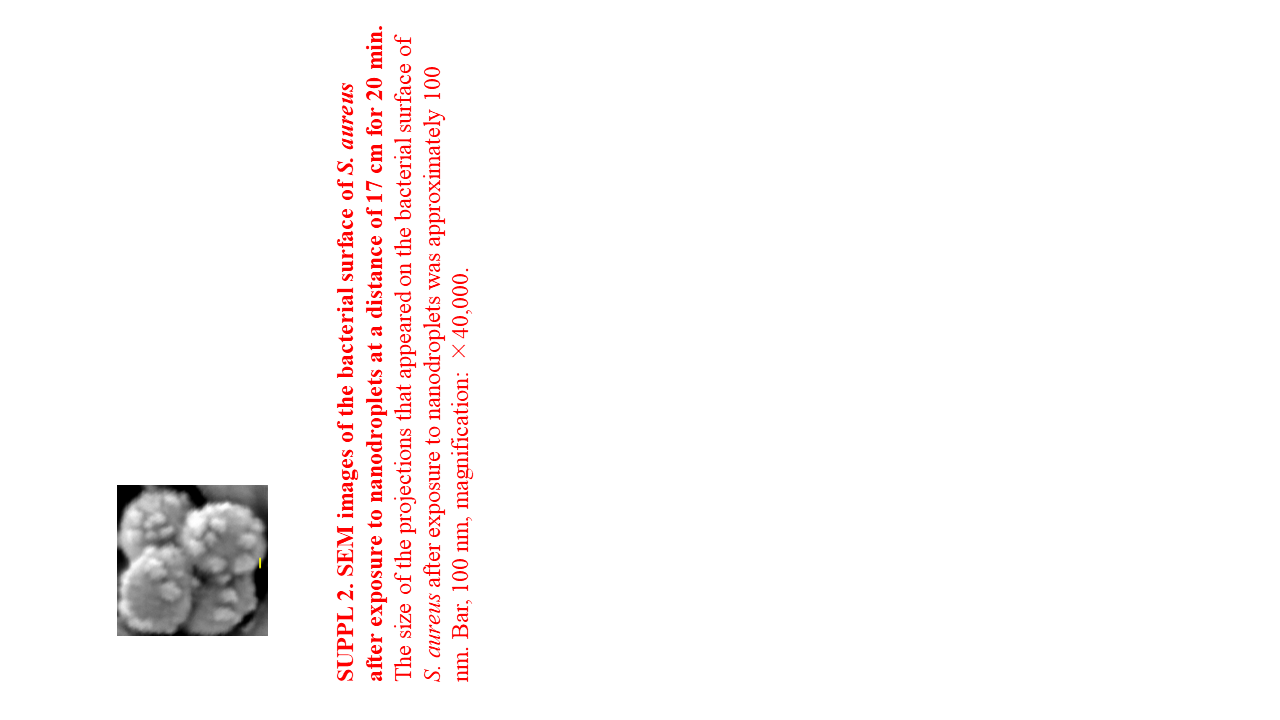

Supplement: Figure S2 — SEM images of the bacterial surface of S. aureus. [file jb.00139-24-s0002.tiff]

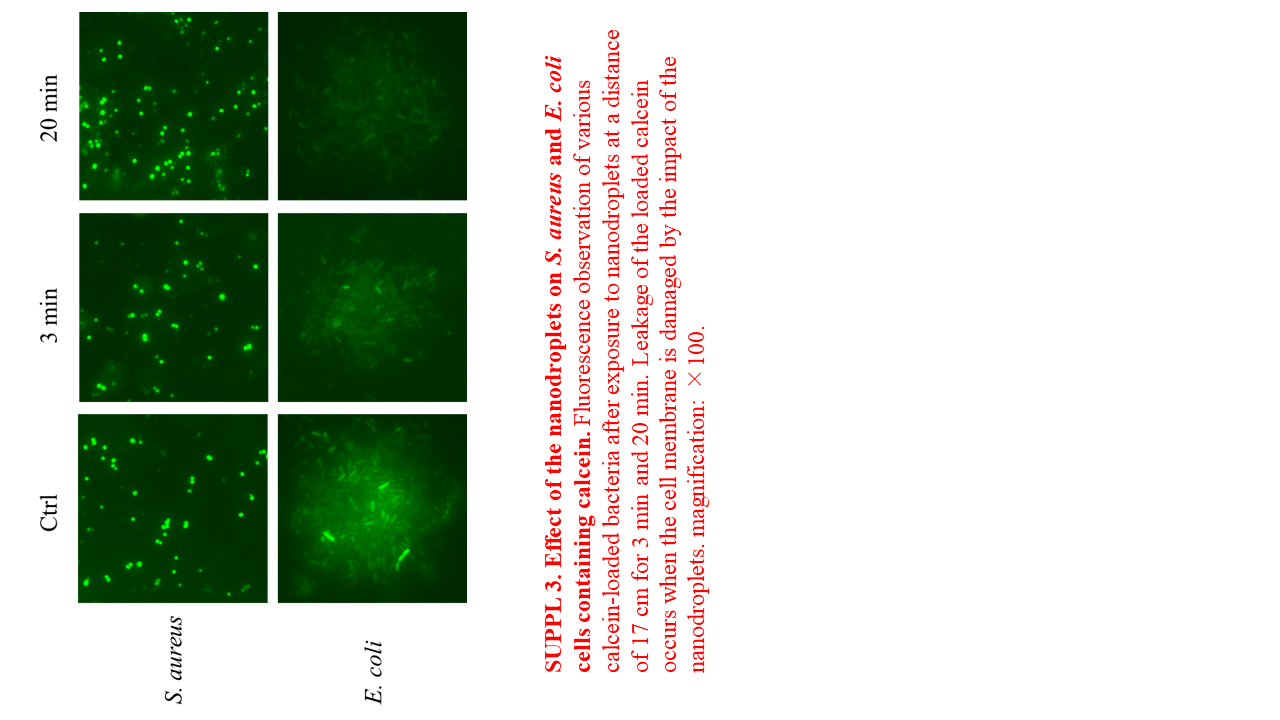

Supplement: Figure S3 — Effect of the nanodroplets on S. aureus and E. coli cells containing calcein. [file jb.00139-24-s0003.tiff]
